# A clinical accuracy study protocol for a saliva-based point-of-care lateral flow test to assess protective immunity to tetanus (TETANUS study)

**DOI:** 10.1136/bmjopen-2026-119202

**Published:** 2026-08-12

**Authors:** K Gokani, SE Faustini, C Tanner, HF Kwok, R Agarwal, Y Takwoingi, Semukunzi H, C Umuhoza, A Richter, J Nyombayire, CM Muvunyi, J Heaney, CA Green

**Affiliations:** 1 School of Chemical Engineering, College of Engineering and Physical Sciences, University of Birmingham, Birmingham, UK; 2 Dept of Infectious Diseases & Tropical Medicine, NIHR/Wellcome Clinical Research Facility, University Hospitals Birmingham NHS Foundation Trust, Birmingham, UK; 3 Clinical Immunology Service,School of Infection, Inflammation and Immunology, College of Medicine and Health, University of Birmingham, Birmingham, England, UK; 4 Department of Applied Health Sciences, School of Health Sciences, College of Medicine and Health, University of Birmingham, Birmingham, UK; 5 University of Rwanda, Kigali, Rwanda; 6 Center for Family Health Research, Kigali, Rwanda; 7 Rwanda Biomedical Centre, Kigali, Rwanda

**Keywords:** Immunity, Immunization Programs, Paediatric infectious disease & immunisation, Pregnant Women, Research Design, Vaccination

## Abstract

**Abstract:**

**Introduction:**

Despite the availability of a safe and effective vaccine, 2000–3000 neonates die of tetanus yearly, predominantly in low- to middle-income countries (LMICs). Inaccurate coverage estimates and vaccination records, together with variability in vaccine response, make identifying individuals who lack protective immunity and may benefit from vaccination difficult. A more direct measure of protective immunity is widely accepted as anti-tetanus IgG concentration at or above 0.1 IU/mL. This study aims to evaluate the performance of a novel saliva-based point-of-care lateral flow test (CIS-IMMUNE Tet) for the binary classification of tetanus protective immune status (protected/unprotected) against WHO threshold-based classification of IgG concentration.

**Methods and analysis:**

This is a diagnostic test accuracy study with a cross-sectional design conducted in Rwanda. A total of 390 participants will be prospectively enrolled through direct invitation by health centres and community health workers using a convenience sampling approach. Participants will be enrolled into four groups: children aged 5–10 years, healthy adults aged 18–25 years, pregnant women and adults aged 18–45 years with known immunosuppression. This sample size was estimated assuming a specificity of 85% based on proof-of-concept data, with a lower bound of the 95% CI no less than 65%, a significance level of 0.05 and 80% power using the exact binomial method (SAS POWER procedure). Protective immunity will be determined by analysis of anti-tetanus toxoid IgG concentrations in serum by ELISA and analysis of saliva samples using the CIS IMMUNE® Tet test. Test performance will be evaluated by estimating sensitivity, specificity, positive and negative likelihood ratios, each with CIs, for detecting protective immunity using the serum reference standard.

**Ethics and dissemination:**

This study protocol was approved by the Rwanda National Ethics Committee (reference RNEC587/2024) and the University of Birmingham (reference ERN_5194-Oct2025). Results will be published in peer-reviewed medical journals and presented at national and international conferences.

**STRENGTHS AND LIMITATIONS OF THIS STUDY**

- This is conducted in a low-resource setting where the test is most likely to be used.
- Seroprotection predictions are based on vaccination coverage estimates and limited data from serosurveys, as there was no Rwanda-specific data, which may affect the precision of the sample size estimate.
- The population that will be assessed is limited to one urban centre in Rwanda, limiting the generalisability of the results.
- This study does not include an evaluation of the cost-effectiveness of this immune diagnostic approach in wider efforts to achieve tetanus control.

## Background

 Tetanus is caused by inoculation of open wounds with the spores of *Clostridium tetani* bacilli, leading to neurotoxin production and the characteristic symptoms of muscle spasms and rigidity.^1 2^ Annual mortality rates from neonatal tetanus alone are estimated as approximately 2000–3000; however, the majority of deaths occur in rural settings, and therefore this is likely an underestimate.^3^ Following disruption to routine vaccination by the COVID-19 pandemic, annual global cases of tetanus have risen from 14 000 in 2020 to 28 000 in 2024.^2^ These cases are concentrated in five countries, three of which are in sub-Saharan Africa.^2^ Abundance of spores in the environment makes avoidance difficult; however, disease prevention through immunisation is highly effective.^2^

Tetanus toxoid-containing vaccines (TTCVs) are designed to safely stimulate the production of systemic antibodies that can bind to and neutralise free tetanus toxin to prevent disease in the central nervous system. Achieving anti-tetanus toxoid antibody at or above 0.1 IU/mL, the concentration in blood needed for clinical protection, requires multiple TTCV doses over time.^4^ WHO guidelines recommend three doses of TTCVs before 6 months of age, followed by three booster doses between 1 and 15 years, with additional doses in pregnancy guided by vaccination history to minimise the risk of adverse reactions associated with pre-existing immunity.^1 5^ TTCVs are commonly administered in the routine infant immunisation schedule as a component of a polyvalent vaccine formulation, such as the pentavalent vaccine, which also includes antigens for protection against diphtheria, pertussis, *Haemophilus influenzae* type b and hepatitis B virus, and alongside other childhood vaccines given concurrently at the same time at 6, 10 and 14 weeks of age for polio, pneumococcus and rotavirus.^6^ Therefore, the absence of tetanus-specific antibody status may act as a proxy for non-receipt of other vaccine antigens.

Despite both routine and additional supplementary immunisation activities (SIA) policies, more than 14 million children missed vaccination doses in 2022, the majority of whom reside in LMICs.^7 8^ Disruptions to immunisation services during the COVID-19 pandemic contributed to a decline in global DTP3 (third dose of diphtheria, tetanus and pertussis vaccine) coverage to 81% in 2021, the lowest level observed since 2008.^9^ Communities with high numbers of zero-dose children, defined as children who have not received a single dose of diphtheria, tetanus and pertussis-containing vaccine (DTP1), also tend to have disproportionately high numbers of under-dosed children (especially those missing DTP3), making these communities high risk for outbreaks of vaccine-preventable diseases (VPDs).^10^

The importance of prioritising sustainable and robust immunisation programmes is reflected in the UN’s Sustainable Development Goal (SDG) 3.8; global access to vaccines for all by 2030.^11^ However, population-level vaccination coverage data does not necessarily reflect individual-level protection needs against disease. Data on TTCV use and population immunisation records in some regions are limited or unreliable. National-level coverage statistics may mask sub-regional inequalities; for example, in East Africa, 67% of children overall were reported to be fully immunised; however, in almost half the sub-regions analysed, this figure was 48% or less.^12^ Even when detailed healthcare records are available, vaccination does not necessarily result in the induction of protective and durable immunity to disease. Heterogeneity in both vaccine seroconversion rates as well as the magnitude and temporal kinetics of antibody concentrations in blood are often impacted by vaccine security (such as the quality of cold-chain custody of the vaccine and vaccine stockouts) and host factors (such as co-morbid disease and childhood malnutrition).^13–17^ Taken together, these limitations highlight the need for a direct, individual-level measure of protective immunity, rather than inference from surrogate data.

Protective immunity to tetanus disease can be measured using in vivo neutralisation tests, modified ELISAs or multiplex assays such as bead-based immunofluorescence assays.^1^ At present, the determination of the immune status of individuals requires patient acceptance of blood tests, skilled phlebotomists, sample transportation systems, centralised and standardised laboratory assays and infrastructure financing. This infrastructure is prohibitively expensive and complex for routine sero-surveillance of the population in resource-poor settings. Community-based point-of-care (POC) tests for anti-tetanus antibodies have been demonstrated to be highly sensitive and specific, including in LMIC settings; however, their use is limited to blood samples.^18^ Salivary anti-tetanus toxin IgG represents a potential non-invasive alternative, with evidence showing good correlation with serum IgG levels.^19 20^

In settings where vaccination records are incomplete and immune responses are heterogeneous, there is currently no reliable, accessible method to determine individual protective immunity at the POC, leading to potential under- or over-vaccination. A rapid, non-invasive diagnostic test could address this gap by enabling real-time assessment of protective immune status. This would allow targeting individuals who are a priority for vaccination and support sero-epidemiology models for measuring the impact of current vaccine policy and plan future public health decisions for tetanus prevention. Successful implementation of POC diagnostics also depends on their acceptability and usability in real-world community and healthcare settings.

This study aims to evaluate the diagnostic performance and acceptability of a novel saliva-based POC lateral flow test (LFT), CIS IMMUNE® TET, for assessing tetanus immune status in a Rwandan population. Rwanda represents a methodologically appropriate setting for this diagnostic accuracy study due to persistent regional and socioeconomic disparities despite high immunisation coverage estimates. For example, coverage ranges from 73% in the Northern Province to 91% in Kigali, with additional heterogeneity within urban populations.^21^ Given the high tetanus burden in East Africa, this setting reflects the intended use environment for the assay and allows evaluation under real-world conditions. Findings are likely to be relevant to similar resource-constrained settings.

The design, development and optimisation of the immunodiagnostic test were performed by the Clinical Immunology Services, University of Birmingham, UK, and will be published separately.

## Methods and analysis

### Trial design and setting

This is a prospective, test accuracy study with a cross-sectional design, incorporating a qualitative component. All study procedures will be conducted at a single facility, the Centre for Family Health Research (CFHR) in Kigali, Rwanda, in collaboration with the Rwanda Biomedical Centre (RBC), the national health implementation agency for Rwanda and the University of Birmingham, UK.

### Recruitment

Sampling will be conducted using a convenience-based approach. Individuals attending health centres or identified through community health workers affiliated with CFHR throughout Rwanda will be directly invited to participate. For the qualitative component, a subset of participants and healthcare workers will be selected using purposive sampling to ensure representation across study groups and perspectives.

### Objectives

#### Primary objective

The primary objective is to assess the diagnostic accuracy of CIS IMMUNE® Tet for identifying tetanus immune status (protected/unprotected), using serum anti-tetanus antibody concentrations≥0.1 IU/mL measured by ELISA as the reference standard.

#### Secondary objectives

- To understand perceptions and acceptability of CIS IMMUNE Tet by healthcare workers and members of the public who participated in the study.
- To explore the relationship between salivary test results, including test line intensity, serum anti-tetanus antibody concentrations and vaccination history.
- To explore factors that underpin variations in serum anti-tetanus toxoid antibody concentration, including age, sex and weight.
- To evaluate the extent to which tetanus antibody status may act as a proxy for immunity to other antigens included in routine vaccination programmes.

### Eligibility

We aim to recruit a total of 390 study participants from the following study groups:

- Group A: Healthy children aged 5–10 years (n=250)
- Group B: Healthy younger adults aged 18–25 years (n=35)
- Group C: Healthy pregnant women (n=30)
- Group D: Adults with known immune suppression (see table 1) aged 18–45 years (n=75).

**Table 1:** List of medical conditions associated with immune suppression for Group D

Haematology disorders	History of haematopoietic stem cell transplantation Acute leukaemia Lymphomas Myelodysplastic syndromes
Autoimmune disorders	Patients on certain biological modifiers, eg, TNF-α blocker, anti-CD20 monoclonal antibody treatments Receiving daily corticosteroid therapy with a dose≥20 mg of prednisone or equivalent for ≥14 days
Malignant solid tumours	Those currently receiving chemotherapy
Primary immunodeficiency disorders	Common variable immunodeficiency Complement deficiency Less severe impairment of antibody production, eg, IgG subclass deficiency, specific antibody deficiency
Secondary immunodeficiency disorders	HIV infection with a CD4 cell count of <200/mm^3^
Renal disorders	Chronic renal failure requiring haemodialysis
Solid organ transplant recipient	All solid organ transplant recipients

CD, cluster of differentiation; HIV, Human Immunodeficiency Virus; TNF-α, Tumor Necrosis Factor-alpha.

These groups were selected to reflect the use case of the test (children aged 5–10 years and pregnant women, who are often targeted by SIA), while balancing the need for sufficient tetanus seronegative individuals to allow robust test evaluation.

Eligibility criteria reflect the study groups outlined in box 1. Exclusion criteria include any condition that might reduce vaccine response (except for Group D) or any condition that may make participation in this study an unacceptable risk to the participant.

Box 1 **Inclusion/exclusion criteria**

**Inclusion criteria – all must be satisfied:**

Able and willing to provide informed consent to take part in the study, either directly or from a parent/guardian, where appropriate

(Group A) Aged 5–10 years inclusive and determined as healthy by a member of the study team

(Group B) Aged 18–25 years inclusive and determined as healthy by a member of the study team

(Group C) Currently pregnant at any stage of pregnancy, prior to receipt of a tetanus booster vaccine in pregnancy and determined as healthy by a member of the study team and safe to provide a blood sample

(Group D) Adults aged 18–45 years with one or more of the medical conditions that may impair immunity.

**Exclusion criteria – none can be satisfied:**

Participants or parents/guardians unwilling or unable to provide informed consent to take part

Unwilling or unable to comply with study procedures

Have a bleeding disorder deemed significant by study doctor

(Groups A, B and C only) Any health condition which, in the opinion of a study physician, could affect immune response to a vaccine, for example known/suspected impairment of immune function (with the exception of the conditions in table 1 for Group D)

### Study procedures

All study procedures can be completed in one visit. All participants will provide informed consent using the latest Rwanda National Ethics Committee (RNEC)-approved combined Study Information Booklet and Informed Consent Form (SIB/ICF) prior to commencement of any study procedures. Participants may provide optional separate consent for future use of their samples in other vaccine research and/or for use of fully anonymised samples by commercial partners. All secondary use will be subject to appropriate ethical approval and institutional oversight, with controlled access procedures in place and clear separation between research and commercial use pathways.

Baseline assessments will include age, sex, country of birth, current and past medical history, body mass and height in Group A (5–10 years), pregnancy history where appropriate, vaccination history, current medications and national identification number. A participant is enrolled following consent and confirmation of eligibility at the point of sample provision.

No formal follow-up is planned. Participants will be informed whether their serum antibody levels are above or below the WHO protective threshold, and those below the threshold will be offered a licensed TTCV dose.

### Safety

The study has minimal risk to participants. Phlebotomy carries a small risk of transient bruising and discomfort.

### Participant samples

Each participant will be asked to provide a peripheral blood sample of up to 5 ml (dependent on age) and one saliva swab (JSHXRT swab, Jiangsu HXRT MD Co., Ltd, China). Samples will be collected according to local and study-specific standard operating procedures.

### Sample processing, storage and analysis

#### Saliva collection, processing and reporting of index test results

**Figure 1:**
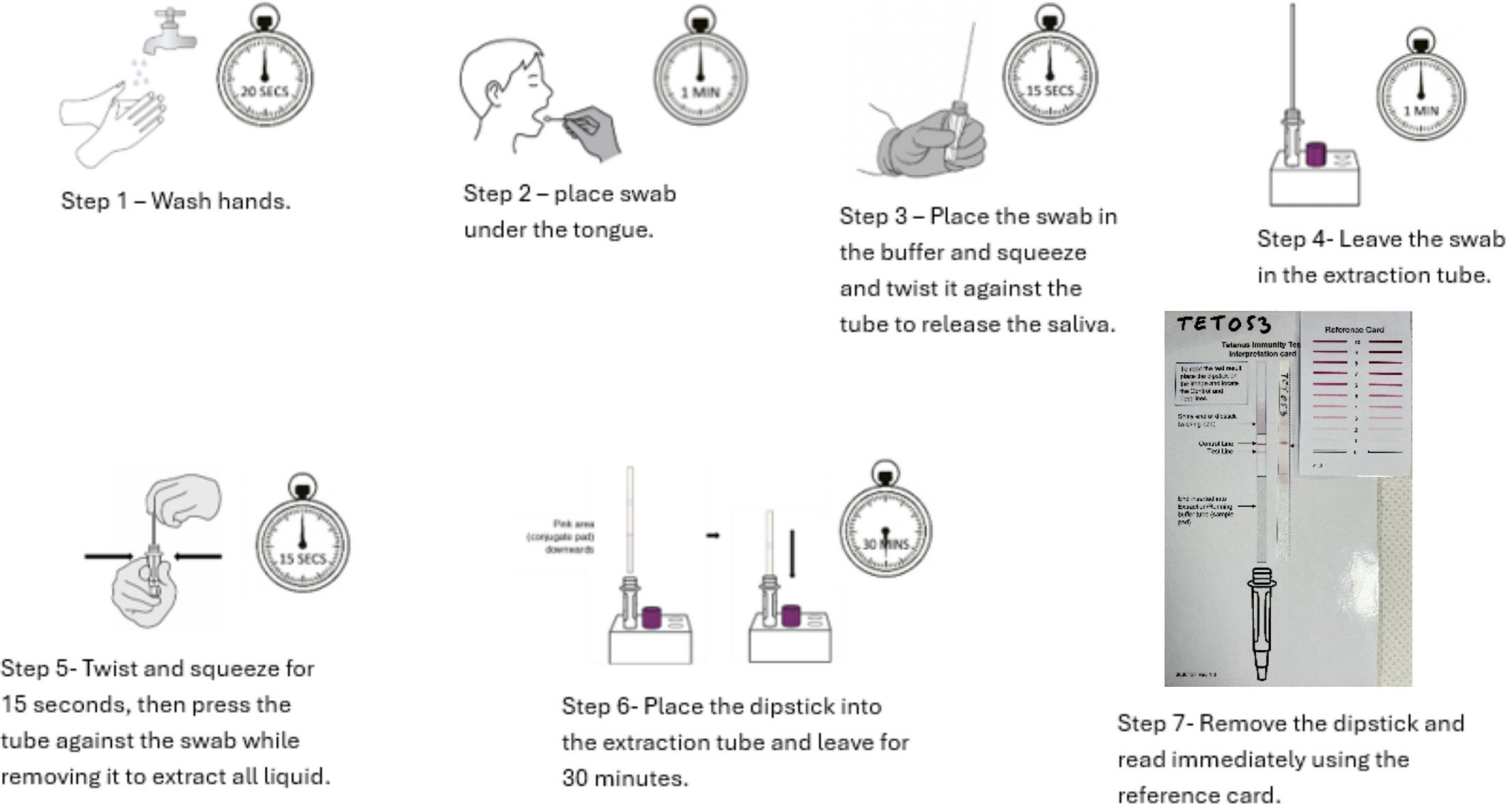
Saliva collection procedure. Each step should be taken immediately after the previous step.

All study staff will receive in-person training on test use and interpretation (outlined in figure 1), with scoring assessed using sample images and benchmarked against experienced University of Birmingham assessors. CIS IMMUNE Tet will be stored at 2–8°C.

Participants will be asked to confirm they have not eaten or drunk (except water) in the 30 minutes prior to sampling. If this criterion is not met, sampling will be delayed until the 30 minute period has elapsed. This restriction is intended to minimise variability in saliva composition and reduce potential contamination that could affect assay performance. Participants will be given the choice to collect their saliva sample themselves under staff supervision or have it collected by a member of staff. Each test will be scored independently by two trained readers (a nurse and a laboratory technician) using a standardised reference card. Results will be photographed at the point of reading so that two additional readers at the University of Birmingham can review with a third reader to resolve any discrepancies in scoring. Scores more than or equal to one will be classed as positive.

#### Serum processing

There will be no storage of cells or DNA. Peripheral blood will be collected in serum clotting tubes and stored at 2–8 °C under continuous temperature monitoring for up to 4–6 hours prior to processing. Serum will be separated by centrifugation at 1600×g for 5 minutes and subsequently heat-inactivated at 56 °C for 60 minutes, with brief vortexing at 30 min to ensure uniform heat distribution. Samples will then be cooled immediately to 4 °C using an ice bath or refrigerator, then stored at −80 °C.

#### Serum analysis

Serum samples will be shipped to the University of Birmingham Clinical Immunology Services on dry ice (UN1845) using an IATA-compliant triple-packaging system. Shipments will be maintained at ≤−20 °C throughout transit, with sufficient dry ice to ensure temperature stability for the full transport duration plus a 24-hour contingency. All packages will be labelled according to IATA PI650/PI954 requirements and transported via a certified cold-chain courier operating validated quality-assurance procedures.

The samples will then be analysed for anti-tetanus toxoid IgG antibody concentration using commercial tetanus IgG ELISA kits (Binding Site, Edgbaston, UK) according to the manufacturer’s instructions. Anti-tetanus toxoid IgG antibody levels≥0.10 IU/mL will be classed as seroprotected. For those who consent, samples will undergo additional testing for other vaccine antigen-specific antibody levels in the EPI schedule using commercially available ELISA assays according to manufacturer instructions.

#### Blinding

Participants will be blinded to the LFT result, as the accuracy of the assay is under evaluation in this study. To maintain blinding, participants will leave the clinic room after providing a saliva sample. The LFT will first be scored by the nurse who collected the sample, followed by independent scoring by a laboratory technician. Laboratory personnel analysing serum samples will be blinded to both the clinical data and the LFT scores.

### Sample storage

Samples will be stored under secure conditions for a maximum of five years. This retention period is considered proportionate to the study aims and allows sufficient time for analysis, validation and audit, in line with standard research governance practices and institutional and ethical requirements.

### Acceptability and feasibility assessment

A subset of participants and healthcare professionals will be invited to take part in focus groups to receive feedback on their perceptions of antibody testing and of using CIS IMMUNE® Tet. A maximum of five participants per group and 10 healthcare professionals will be recruited. The proposed sample size has been pragmatically determined, balancing the exploratory qualitative aims of the study with feasibility considerations, including time and resources, while still enabling collection of a range of relevant perspectives.

Focus groups will explore attitudes to tetanus infection, vaccination, blood tests as compared with saliva tests, participation in clinical research and use of a test to determine immune status and need for vaccination. Sessions will take place in groups of 5–15 individuals and will be led by a trained qualitative researcher in Kinyarwanda using an RNEC-approved, semi-structured topic guide. Reflexivity will be maintained through regular team discussions to reflect on interviewer influence on data collection and interpretation. Facilitators will not be involved in participants’ clinical care, minimising potential bias or influence on responses.

Participants and healthcare workers will be interviewed and analysed separately using separate topic guides. Participants from separate groups may be interviewed together. Audio recordings will be transcribed by a designated researcher, with a second reviewer checking the transcription against the original recording for accuracy. Transcripts will then be translated, with a second reviewer verifying the translation against the original transcript. No identifiable information will be included in translated documents. Any discrepancies will be documented and resolved by consensus between the two transcribers or translators.

### Sample size

The sample size was calculated to ensure adequate precision in estimating the sensitivity and specificity of CIS IMMUNE® Tet against a serum reference standard, with primary emphasis on specificity.

Assuming a specificity of 85% based on proof-of-concept data, with a lower bound of the 95% CI no less than 65%, a significance level of 0.05 and 80% power, at least 35 participants below the WHO protective threshold are required. This lower-bound confidence limit was selected due to limited seroprevalence data in this region and a possibly low number of seronegative individuals. The sample size was calculated using the exact binomial method in the SAS POWER procedure.^22^

The total sample size was therefore driven by the need to obtain 35 unprotected participants. To obtain this number of unprotected participants, an overall protection (seropositivity) rate of 90% was assumed based on vaccine coverage estimates and serosurveys from East Africa.^23 24^ As no seroprevalence data are available for Rwanda, this estimate was informed by proxy indicators, including DTP3 coverage of 94–98% since 2015.^23^ Under this assumption, using a stratified recruitment strategy, a total sample size of 350 participants would yield approximately 33–50 individuals below the WHO protective threshold, primarily through targeted recruitment of immunosuppressed individuals (table 2). As true serostatus will be unknown until recruitment has concluded, the group-specific targets are fixed.

**Table 2:** Estimated distribution of protective immunity and expected number of participants below the WHO protective threshold by study group. Estimated proportions protected were derived from serosurveys in East Africa or vaccine coverage estimates. Seroprotection in those with known immunosuppression was estimated from studies in both low- and high-income settings, as only data related to HIV were available from LMICs

Group	Estimated proportion seroprotected	Target number	Estimated number below the protective threshold
Group A: Healthy children aged 5–10 years	≥97%^23^	250	7–8
Group B: Healthy younger adults aged 18–25 years	≥97%^32^	35	1–2
Group C: Healthy pregnant women	≥90%^33^	30	3
Group D: Adults with known immune suppression aged 18–45 years	50–70%^34–36^	75	22–37

The remaining 315 participants with protective immunity provide greater than 80% power to estimate sensitivity, assuming a true sensitivity of at least 70%, with the lower bound of the 95% CI no less than 60%. The precision of the sensitivity estimate is therefore conditional on the achieved prevalence of protective immunity within the study population. To account for potential withdrawal, missing data and invalid or indeterminate test results affecting the completeness of the index test and/or reference standard measurements, the total sample size was increased by 10%, resulting in a final recruitment target of 390 participants.

### Statistical analysis plan

All analyses will be conducted according to a pre-specified statistical analysis plan using GraphPad Prism (V.10), RStudio (RStudio v2026.01.0-392) and Microsoft Excel 365 (Microsoft, V.2508). A two-sided significance level of 0.05 will be used throughout.

#### Primary analysis

The primary analyses will include the whole study population with available index test and reference standard results. Indeterminate or invalid index test results will be reported explicitly and treated as test failures for the primary analysis.

To address the primary objective (diagnostic accuracy of CIS IMMUNE® Tet), the performance of the index test will be evaluated against the reference standard (ELISA-based seroprotection threshold ≥0.1 IU/mL).

Sensitivity, specificity and positive and negative likelihood ratios will be estimated from 2×2 contingency tables, with corresponding 95% CIs calculated using exact (Clopper-Pearson) methods.^25 26^

#### Secondary analyses

Secondary analyses are not powered to detect statistically significant effects and will be considered exploratory and hypothesis-generating.

##### Acceptability and perceptions

Data collection and analysis from the focus groups will be grounded in the theoretical perspective of interpretivism as a framework for exploring the meanings, beliefs and values that guide people’s actions and decision-making. Data will be analysed using reflexive thematic analysis as outlined by Braun and Clark.^27 28^ This involves a six-phase non-linear recursive process.^28^

##### Factors affecting salivary test results

Inter-reader agreement in LFT line intensity and LFT results will be assessed using percentage agreement and Gwet’s AC index, with 95% CIs. The relationship between LFT line intensity and serum anti-tetanus IgG concentration will be explored using Spearman’s rank correlation, where the correlation coefficient will be reported.

##### Agreement with vaccination history

Agreement between seroprotective status as measured by the LFT or ELISA and reported vaccination history will be assessed using percentage agreement, with 95% CIs.^29–31^ Given known limitations in vaccination records via vaccination cards and maternal recall leading to potential misclassification, this analysis will be interpreted cautiously and considered exploratory.

##### Determinants of antibody levels

Variation in serum anti-tetanus IgG concentrations will be explored using a multivariable linear regression model including age, sex, malnutrition status, study group, vaccination history and immunosuppression status as covariates.

##### Relationship with other vaccine antigens

The ability of tetanus seronegativity to identify seronegativity to other antigen-specific antibodies used in infant polyvalent vaccines (for diphtheria and Hib) will be assessed using positive and negative percent agreement derived from 2×2 tables, with 95% CIs.^29^ As these analyses compare test results without a reference standard, they will be treated as agreement analyses rather than diagnostic accuracy assessments.

### Multiplicity and inferential framework

No formal adjustment for multiple comparisons will be applied to secondary or exploratory analyses. These analyses are not powered for hypothesis testing and will be interpreted as exploratory, with findings used to generate hypotheses for future studies. Emphasis will be placed on effect sizes and CIs rather than p-values.

### Missing data

No formal imputation will be performed. The primary analysis will include only participants with complete paired index test and reference standard results. The extent and reasons for missing data will be reported.

### Data management plan

All data are pseudonymised at source before processing at laboratory facilities. At screening, all participant data and samples will be labelled with a unique study-specific participant number. Personally identifiable information (eg, name, contact details) will be stored separately from study data. The linkage key connecting participant identities to study IDs will be held securely at CFHR in a restricted-access file, with access limited to authorised study staff only and protected by password and encryption. This key will not be transferred outside CFHR.

Study data will be collected via an electronic case report form (eCRF) in REDCap by trained and authorised personnel. Data will be stored on secure, encrypted servers at RBC, with role-based, password-controlled access limited to authorised study team members. Data quality will be ensured through REDCap validation checks, including range checks, mandatory fields and logic constraints at the point of data entry and audit trails. Prior to analysis, datasets will undergo systematic data cleaning, including checks for completeness, consistency, outliers and logical errors. Queries will be resolved against source data where required. Missing data patterns will be assessed descriptively.

Audio files will be stored on a secure server at CFHR, and only pseudonymised, transcribed and translated files of the focus groups will be shared outside of CFHR.

No directly identifiable data will be shared with collaborators outside of CFHR. Any data transferred for analysis will be pseudonymised. Re-identification will only occur where necessary for study conduct (eg, follow-up or safety reporting) and will be performed solely by authorised staff.

## Discussion

This study aims to evaluate the real-world clinical accuracy of a novel, saliva-based, POC lateral flow test to determine an individual’s immune status to tetanus in Rwanda.

Tetanus remains a significant cause of mortality in LMICs. Fourteen countries have yet to reach maternal and neonatal tetanus elimination (MNTE), 10 of which are in sub-Saharan Africa.^5^ Antenatal screening has already been identified as a tool in achieving MNTE;^5^ however, a POC test to detect tetanus IgG above the WHO immune correlate of protection would allow use in all ages to support targeted and more efficient vaccine utilisation. This test could be used to personalise vaccine decision-making, ensuring only those who need vaccination receive them, possibly reducing the resources required to deliver SIA. This test would require only a saliva sample and the study will inform if this approach is acceptable to participants and caregivers. As tetanus is administered as part of a pentavalent vaccine, this test could also be used as a surrogate marker for possible immunity gaps to other pathogens included in polyvalent vaccine formulations.

### Patient and public involvement statement

There was no public involvement in the development of the research question or study design. However, the qualitative component of the study (planned semi-structured interviews or focus groups) with study participants and healthcare workers ensures local engagement and perspectives are integrated into the research project.

### Ethics and dissemination

This study protocol has been reviewed and approved by the RNEC (reference RNEC 587/2024) and the University of Birmingham (reference ERN_5194-Oct2025). The study will be conducted in accordance with the principles of the Declaration of Helsinki and relevant regulations of the International Conference on Harmonisation Guidelines for Good Clinical Practice. The sponsor is the University of Birmingham, which provides indemnity or compensation for harm arising as a consequence of the research subjects’ participation in the study.

Participation in the trial is strictly voluntary, and withdrawal can occur at any point in the trial. Participants will be reimbursed a small fee as compensation for expenses incurred as part of taking part in the study.

Results will be published in a peer-reviewed medical journal and presented at national and international conferences. All publications (eg, manuscripts, abstracts, oral/slide presentations and book chapters) based on this study will be reviewed by each investigator prior to submission.
