# Supplementary material for: A clinical accuracy study protocol for a saliva-based point-of-care lateral flow test to assess protective immunity to tetanus (TETANUS study)
Source: BMJ Open. 2026 Aug 12;16(8):e119202. doi: 10.1136/bmjopen-2026-119202 (PMC13475110; doi:10.1136/bmjopen-2026-119202)
Supplement: Supplementary file 1 [file bmjopen-2026-119202.reviewer_comments.pdf]

PEER REVIEW HISTORY

BMJ Open publishes all reviews undertaken for accepted manuscripts. Reviewers are asked to complete a checklist review form and are provided with free text boxes to elaborate on their assessment. These free text comments are reproduced below.

ARTICLE DETAILS

Title (Provisional)

Tetanus antibody detection in saliva (TETANUS) study -development of novel diagnostics that use point-of-care lateral flow testing technology for non-invasive, individual assessment of antibody protection to tetanus and vaccine need: A clinical accuracy study protocol

Authors

Gokani, K; Faustini , SE; Tanner, C; Kwok , HF; Agarwal, R; Takwoingi, Y; H, Semukunzi; Umuhoza, C; Richter, A; Nyombayire, J; Muvunyi, CM; Heaney, J; Green, CA

VERSION 1 - REVIEW

**Reviewer**	**1**
**Name**	**Santos, João**
**Affiliation**	**National Health Institute Doutor Ricardo Jorge, Department of Epidemiology**
**Date**	**23-Apr-2026**
**COI**	**None**

Thank you for the opportunity to review this manuscript. The study will explore the performance of a novel point-of-care, saliva-based lateral flow test for detecting protective immunity to tetanus. Considering the ongoing burden of neonatal tetanus in low- and middle-income settings and the challenges associated with identifying individuals in need of supplementary immunisation, the evaluation of rapid, field-based diagnostic approaches is of considerable public health and epidemiological interest.

Below, I outline comments intended to enhance the clarity, structure, and methodological transparency of the protocol.

Title

The title is informative and captures the key elements of the study; however, it could be improved for clarity, conciseness, and readability, as it is somewhat lengthy and contains redundant phrasing (e.g., overlapping descriptions of tetanus antibody detection and protection, and repeated reference to diagnostic technology). Please improve.

Suggestions: “A clinical accuracy study protocol for a saliva-based point-of-care lateral flow test to assess protective immunity to tetanus (TETANUS study)” or “Saliva-based point-of-care testing for tetanus immunity: a clinical accuracy study protocol (TETANUS study)”

ABSTRACT

"Waning immunity, limited access to vaccines, poor access to care and other challenges make the identification of individuals in need of supplementary immunisation efforts difficult." - This sentence is conceptually misleading, as waning immunity, limited access to vaccines, and poor access to care do not impede identification of individuals requiring supplementary immunisation; instead, they increase the proportion of individuals potentially in need while the difficulty in identification arises from limited access to reliable vaccination records and immune status assessment. Please revise.

The study aim is imprecisely defined, as the phrase “real-time, in-field determination of immune status” is vague and does not specify whether the test provides a qualitative or quantitative assessment. Also the intended use of the test should be more clear, as “determination of immune status” is not equivalent to assessing protective immunity; individuals may have detectable antibodies without reaching concentrations considered protective.

The reference to the WHO correlate of protection (0.1 IU/mL) is introduced abruptly after the aim and should be presented earlier to provide the basis for the study objective. Additionally, the phrase “provide a signal of detection” is imprecise and should be replaced with clearer wording (e.g., “detect anti-tetanus toxoid IgG at or above…”).

The recruitment strategy (“direct invitation materials”) is vague and insufficiently described. Please improve.

The description of the study groups is vague, being unclear what study groups are defined for secondary analyses. Please improve.

The statistical analysis plan is incomplete. While sensitivity, specificity, and likelihood ratios are mentioned, there is no information on sample size justification, handling of uncertainty (e.g., confidence intervals), or how discordant results will be addressed.

INTRODUCTION

Please follow the nomenclature and structure of BMJ Open for Protocols. (https://bmjopen.bmj.com/pages/authors#protocol).

Foundational information on tetanus pathophysiology (lines 58–66) is disproportionately detailed for a protocol and detracts from the focus on the research problem. This section should be substantially condensed, retaining only information directly relevant to the study rationale.

The references are outdated (e.g., Roper MH, et al. 2007) and some are not traceable (e.g., ref 5 to ref 7). The authors need to substantially improve their references, both in terms of reflecting current knowledge and in terms of traceability.

"Communities with high numbers of zero dose children, defined by GAVI" - Please define the meaning of GAVI.

"...(DTP1)(9), are also enriched with under dosed children..." - it sounds off. “Enriched with under-dosed children” suggests children are somehow a benefit to the community, which isn’t what you mean (hopefully). Please revise.

The narrative lacks clear progression and coherence. Key themes (disease burden, vaccination coverage, immunity gaps, and health system limitations) are presented in a fragmented manner without a logical flow toward the study rationale. Several statements are repetitive, particularly regarding vaccine access, waning immunity, and vulnerable populations.

Authors could reorganise the background into a clear structure: (1) disease burden, (2) vaccination strategy and limitations, (3) gap between vaccination and protective immunity, (4) limitations in current assessment methods, (5) rationale for the proposed diagnostic. There is no reason for the Study rationale to not be integrated in the introduction (Introduction: explain the rationale for the study and what evidence gap it may fill. Appropriate previous literature should be referenced, including relevant systematic reviews.).

There are also structural and formatting issues, including sentence fragmentation, misplaced numbering, and interruption of text flow, which reduce readability.Eliminate redundant statements and streamline language throughout.

The link between existing gaps (e.g., incomplete records, heterogeneous immune responses) and the need for a point-of-care diagnostic is not articulated. The rationale for the study is therefore underdeveloped and lacks a clear statement of the specific problem the proposed test addresses.

"Rwandan population" - The authors should strengthen the manuscript by further highlighting the relevance of conducting this study in the Rwandan population, particularly as it represents a setting where the test is most likely to be used and where its real-world performance can be meaningfully assessed. Its relevance as a proxy for similar resource-constrained settings and the implications for generalisability should be highlighted.

Currently, the Background and Study Rationale sections appear to address similar themes but function as largely separate components, rather than forming a cohesive narrative that logically leads to the study objective and clearly justifies its importance.

Methods and analysis

* Trial design and setting

"This is a cross-sectional, prospective, test accuracy study" - this phrasing is technically ambiguous and should be clarified because “cross-sectional” and “prospective” are not naturally compatible unless carefully defined. More precise - “This is a prospective diagnostic test accuracy study with cross-sectional sampling.”

The “Trial design and setting” subsection contains an inappropriate mixture of study design description, site description, and epidemiological background. The inclusion of national vaccination coverage data and refugee population statistics is not clearly linked to study design or analytical purpose and appears descriptive rather than methodologically justified. Please revise.

The setting description is incomplete in methodological terms. It does not specify catchment population, inclusion pathways (e.g. community vs facility-based recruitment), or whether the setting is intended to be representative or convenience-based. Please revise.

The rationale for selecting the study setting is not explicitly integrated into the design justification and remains epidemiological narrative rather than methodological argumentation. Please revise.

* Aims and objectives

The “Aims and objectives” section lacks clear alignment with the stated study design. The primary objective is appropriate for a diagnostic accuracy study; however, the secondary objectives are heterogeneous and extend beyond diagnostic accuracy (including acceptability, immunological correlation analyses, and multi-pathogen antibody profiling). These are not clearly justified within the described study design and appear methodologically disparate. Please revise.

The objectives are not prioritised or structured according to primary and secondary endpoints, and there is no indication of how each objective will be operationalised within the study design framework. Please revise.

Overall, the section requires restructuring to separate study design, setting justification, and objectives, and to ensure alignment between stated design, data collection framework, and planned analyses.

* Study interventions

The heading “Study interventions” is misleading, as the study does not involve an intervention in the clinical trial sense. The content instead describes consent procedures, baseline data collection, and sample handling, which should be relocated to a “Study procedures” or “Data collection” section.

"using the latest RNEC-approved" - define RNEC

The statement that “a participant is enrolled at the point of sample provision” is imprecise and does not define eligibility confirmation, consent completion, or inclusion criteria in operational terms.

* Saliva collection, processing and reporting of index test results

The description of the index test procedure is overly narrative and insufficiently standardised. Key operational details are missing, including swab type specification, storage conditions if needed, and time limits between collection, processing, and test initiation.

The pre-analytical instructions (“no eating or drinking except water for 30 minutes”) are stated without justification or reference to standardisation criteria, and it is unclear whether adherence will be monitored.

The saliva collection procedure is not fully specified in terms of training requirements for staff versus self-collection, nor is there clarification on whether both methods will be used interchangeably or under defined conditions.

The interpretation process lacks adequate blinding procedures. Although dual scoring is described, there is no explicit statement regarding blinding of scorers to clinical data or reference standard results, nor is inter-rater reliability assessment planned or described.

The use of a semi-quantitative 0–10 intensity scale is introduced without validation, calibration procedures, or explanation of how this variable will be used in analysis, raising concerns about measurement validity and reproducibility.

The statement that participants will be blinded to LFT results is methodologically unclear, as it is not explained how blinding will be maintained in practice, particularly given immediate interpretation at point of care.

* Laboratory analysis plan

The laboratory analysis plan lacks sufficient methodological detail to ensure reproducibility and clarity in a diagnostic accuracy protocol.

The designation of “multiplex bead-based assay and ELISA” as reference standards is not adequately justified or operationalised. It is unclear whether both assays will be used independently, in parallel, or hierarchically, and no algorithm is provided for resolving discordant results. The reference standard definition therefore remains ambiguous.

The description of serum processing (clotting, extraction, heat-inactivation) is incomplete in key procedural aspects, including timing, centrifugation parameters, storage temperatures, and chain-of-custody procedures prior to shipment.

The shipping process to the University of Birmingham is stated without specifying transport conditions (e.g. temperature control, duration limits, quality assurance procedures), which is critical for antibody stability.

The blinding statement is insufficiently detailed, as it does not clarify whether laboratory personnel are also blinded to clinical data and index test results beyond the stated assessor role.

The inclusion of additional antibody testing for other vaccine antigens is described without clear linkage to predefined objectives or statistical analysis plans, raising concerns about exploratory data collection without analytical framework.

* Sample storage

The term “pseudo-anonymisation” is used inconsistently with standard research governance terminology, and no clear description of coding, re-identification safeguards, or data linkage procedures is provided.

The duration of sample storage (5 years) is stated without justification or reference to regulatory or ethical requirements.

Consent options for secondary use of samples, including commercial use, are described without clarification of governance framework, oversight, or separation between research and commercial pathways.

* Acceptability and feasibility assessment

This is insufficiently defined methodologically. The sample size (10–20 participants and 10–20 healthcare professionals) is arbitrary and not justified. Recruitment strategy, sampling method, and saturation criteria are not specified.

Focus group structure lacks clarity regarding stratification, group composition, and whether participant and healthcare professional groups are analysed separately.

The description of qualitative methods is limited and does not specify interview guide development, data coding framework, or analytical approach (e.g. thematic analysis framework).

The role of the facilitator is described in general terms without specifying training standards, reflexivity procedures, or potential influence on data collection.

Audio recording and transcription are stated without mention of translation validation, data anonymisation procedures during transcription, or quality control of transcripts.

* Sample size

The derivation of the primary sample size is presented in a fragmented manner, with unclear separation between assumptions for sensitivity and specificity. The calculation logic is difficult to follow, and the link between statistical inputs (specificity, confidence limits, power) and the final sample size is not transparently justified.

The use of an assumed 90% protection rate is not supported with justification specific to the study population, and its applicability across heterogeneous subgroups is not addressed.

The estimation of 35 unprotected participants as the basis for specificity is insufficiently justified for robust precision requirements, and the rationale for selecting a lower confidence limit of 65% is not explained.

The power calculation for sensitivity is described narratively rather than formally, and does not clearly specify whether it is powered independently or conditionally on the assumed prevalence distribution.

The stratified recruitment plan across four groups is not integrated into the statistical justification. It is unclear how stratification contributes to achieving the required number of unprotected participants, and whether recruitment targets are fixed or adaptive.

The subgroup assumptions (e.g. ≥97% protection in children and young adults, 50–70% in immunosuppressed adults) are stated without supporting empirical justification or reference to data sources.

The statement that 42 participants will be below the protective threshold is inconsistently derived and does not reconcile clearly with earlier calculations based on 35 unprotected participants.

The total sample size of 390, including a 10% attrition allowance, is presented without specifying whether attrition is expected to affect both index and reference standard completeness equally, or how missing data will be handled analytically.

Overall, the sample size section lacks transparency, reproducibility, and clear linkage between prevalence assumptions, subgroup structure, and diagnostic accuracy precision requirements.

* Statistical analysis

The distinction between primary and exploratory analyses is not clearly operationalised, and no formal statistical framework is provided to define how exploratory subgroup analyses will be controlled for multiplicity or interpreted in relation to the primary endpoint.

The description of categorical variable analysis is generic and does not specify exact statistical methods, confidence interval approaches, or handling of clustering across predefined study groups.

The primary diagnostic accuracy measures (sensitivity, specificity, likelihood ratios) are listed without specification of calculation methods, confidence intervals, or threshold definitions for classification of index and reference tests.

There is no description of how indeterminate or invalid index test results will be handled in the analysis, nor whether analyses will follow an intention-to-diagnose framework.

Secondary accuracy analyses extend beyond the stated primary diagnostic objective and include multiple antigen targets (diphtheria, HIB) without justification of statistical power, multiplicity control, or pre-specification of hypotheses.

The use of “positive and negative percent agreement” is not clearly justified in relation to established diagnostic accuracy metrics, and its interpretation relative to sensitivity/specificity is not clarified.

The proposed correlation analysis between test line intensity and antibody concentration is not methodologically specified, including the choice of correlation metric, treatment of non-linear relationships, or any planned transformation or standardisation of variables. In addition, its validity is questionable given that line intensity is derived from subjective visual assessment by operators rather than an objective, instrument-based measurement, introducing potential inter- and intra-observer variability that is not accounted for in the current plan.

The comparison with vaccination history is included without addressing known limitations of vaccination record accuracy or misclassification bias.

The data management plan is largely procedural and lacks detail on data validation, audit trails, handling of missing data, and data cleaning procedures prior to analysis.

The description of pseudo-anonymisation is inconsistent with standard terminology and does not clarify whether re-identification is possible or how linkage keys are managed.

No statistical software, versioning, or reproducibility framework is specified for analysis.

Overall, the section lacks a coherent, pre-specified analytical strategy linking objectives to statistical methods, and includes multiple secondary analyses without clear prioritisation, adjustment for multiplicity, or defined inferential framework.

The protocol has clear potential but requires substantial rethinking and restructuring, with improved clarity and methodological detail, to make it adequately informative and reproducible for application by other researchers.

VERSION 1 - AUTHOR RESPONSE

Reviewer: 1

Dr. João Santos, National Health Institute Doutor Ricardo Jorge, NOVA National School of Public Health

Comments to the Author:
Thank you for the opportunity to review this manuscript. The study will explore the performance of a novel point-of-care, saliva-based lateral flow test for detecting protective immunity to tetanus. Considering the ongoing burden of neonatal tetanus in low- and middle-income settings and the challenges associated with identifying individuals in need of supplementary immunisation, the evaluation of rapid, field-based diagnostic approaches is of considerable public health and epidemiological interest.

Below, I outline comments intended to enhance the clarity, structure, and methodological transparency of the protocol.

Title

1. The title is informative and captures the key elements of the study; however, it could be improved for clarity, conciseness, and readability, as it is somewhat lengthy and contains redundant phrasing (e.g., overlapping descriptions of tetanus antibody detection and protection, and repeated reference to diagnostic technology). Please improve.

   Suggestions: "A clinical accuracy study protocol for a saliva-based point-of-care lateral flow test to assess protective immunity to tetanus (TETANUS study)" or "Saliva-based point-of-care testing for tetanus immunity: a clinical accuracy study protocol (TETANUS study)"

Thank you for the feedback, the title has been updated with your suggestion.

ABSTRACT

2. "Waning immunity, limited access to vaccines, poor access to care and other challenges make the identification of individuals in need of supplementary immunisation efforts difficult." - This sentence is conceptually misleading, as waning immunity, limited access to vaccines, and poor access to care do not impede identification of individuals requiring supplementary immunisation; instead, they increase the proportion of individuals potentially in need while the difficulty in identification arises from limited access to reliable vaccination records and immune status assessment. Please revise.

Thank you, this has been corrected to reflect the impact on immunity rather than identification of those lacking immunity (lines 26-27).

3. The study aim is imprecisely defined, as the phrase "real-time, in-field determination of immune status" is vague and does not specify whether the test provides a qualitative or quantitative assessment. Also the intended use of the test should be more clear, as "determination of immune status" is not equivalent to assessing protective immunity; individuals may have detectable antibodies without reaching concentrations considered protective.

Thank you, we have rewritten the aim to increase clarity that the LFT provides a qualitative result of protective immunity (lines 28-31).

4. The reference to the WHO correlate of protection (0.1 IU/mL) is introduced abruptly after the aim and should be presented earlier to provide the basis for the study objective. Additionally, the phrase "provide a signal of detection" is imprecise and should be replaced with clearer wording (e.g., "detect anti-tetanus toxoid IgG at or above…").

Thank you, the correlate of protective has been introduced earlier (line 27-28), and the phrase “provide a signal of detection” has been changed (lines 28-31).

5. The recruitment strategy (“direct invitation materials”) is vague and insufficiently described. Please improve.

The recruitment strategy has been expanded to provide a clear description of participant identification and approach, including the use of community health workers and health centre engagement (lines 34-35).

6. The description of the study groups is vague, being unclear what study groups are defined for secondary analyses. Please improve.

A clear description of the study groups has now been included in the abstract (lines 35-37).

7. The statistical analysis plan is incomplete. While sensitivity, specificity, and likelihood ratios are mentioned, there is no information on sample size justification, handling of uncertainty (e.g., confidence intervals), or how discordant results will be addressed.

The statistical analysis plan has been completed with details of the sample size calculations and a more thorough summary of the analysis plan (lines 37-43).

INTRODUCTION

8. Please follow the nomenclature and structure of BMJ Open for Protocols. (https://bmjopen.bmj.com/pages/authors#protocol).

   Foundational information on tetanus pathophysiology (lines 58–66) is disproportionately detailed for a protocol and detracts from the focus on the research problem. This section should be substantially condensed, retaining only information directly relevant to the study rationale.

This section has been substantially condensed to a single sentence, retaining only information directly relevant to the study rationale and ensuring appropriate focus on the research question (lines 59-60).

9. The references are outdated (e.g., Roper MH, et al. 2007) and some are not traceable (e.g., ref 5 to ref 7). The authors need to substantially improve their references, both in terms of reflecting current knowledge and in terms of traceability.
   "Communities with high numbers of zero dose children, defined by GAVI" - Please define the meaning of GAVI.

The references have been corrected, and older references have been replaced where appropriate. As a result, GAVI is no longer referenced.

10. "...(DTP1)(9), are also enriched with under dosed children..." - it sounds off. “Enriched with under-dosed children” suggests children are somehow a benefit to the community, which isn’t what you mean (hopefully). Please revise.

Thank you for highlighting this, that was not the intended meaning. This has been updated to include more appropriate wording (tend to have disproportionately high numbers of..) (line 86).

11. The narrative lacks clear progression and coherence. Key themes (disease burden, vaccination coverage, immunity gaps, and health system limitations) are presented in a fragmented manner without a logical flow toward the study rationale. Several statements are repetitive, particularly regarding vaccine access, waning immunity, and vulnerable populations.
    Authors could reorganise the background into a clear structure: (1) disease burden, (2) vaccination strategy and limitations, (3) gap between vaccination and protective immunity, (4) limitations in current assessment methods, (5) rationale for the proposed diagnostic. There is no reason for the Study rationale to not be integrated in the introduction (Introduction: explain the rationale for the study and what evidence gap it may fill. Appropriate previous literature should be referenced, including relevant systematic reviews.).
    There are also structural and formatting issues, including sentence fragmentation, misplaced numbering, and interruption of text flow, which reduce readability. Eliminate redundant statements and streamline language throughout.

    Currently, the Background and Study Rationale sections appear to address similar themes but function as largely separate components, rather than forming a cohesive narrative that logically leads to the study objective and clearly justifies its importance.

Thank you for the guidance and feedback, the introduction has been restructured to follow the suggested order. We have also reviewed the text to remove repetition and improve the flow and readability. The background and study rationale have been combined. There is a systematic review of blood-based tetanus POC LFTs, which has been referenced (number 18, Low et al).

12. The link between existing gaps (e.g., incomplete records, heterogeneous immune responses) and the need for a point-of-care diagnostic is not articulated. The rationale for the study is therefore underdeveloped and lacks a clear statement of the specific problem the proposed test addresses.

The introduction has been revised to explicitly articulate the link between incomplete vaccination records, heterogeneity in immune responses, and the lack of a practical method to determine individual protective immunity at the point of care. The rationale for the study has been strengthened to clearly define the unmet need for a rapid, non-invasive diagnostic to inform vaccination decision-making (lines 90-101).

13. "Rwandan population" - The authors should strengthen the manuscript by further highlighting the relevance of conducting this study in the Rwandan population, particularly as it represents a setting where the test is most likely to be used and where its real-world performance can be meaningfully assessed. Its relevance as a proxy for similar resource-constrained settings and the implications for generalisability should be highlighted.

The manuscript has been revised to strengthen the justification for conducting the study in the Rwandan population. Additional text now highlights Rwanda as a representative resource-constrained setting where point-of-care diagnostics are most needed, and where real-world performance can be meaningfully evaluated. The implications for generalisability to similar settings have also been explicitly articulated (line 121-126).

Methods and analysis

* Trial design and setting

14. "This is a cross-sectional, prospective, test accuracy study" - this phrasing is technically ambiguous and should be clarified because “cross-sectional” and “prospective” are not naturally compatible unless carefully defined. More precise - “This is a prospective diagnostic test accuracy study with cross-sectional sampling.”

Thank you for the suggestion. We have clarified the study design and recruitment strategy (line 131).

15. The “Trial design and setting” subsection contains an inappropriate mixture of study design description, site description, and epidemiological background. The inclusion of national vaccination coverage data and refugee population statistics is not clearly linked to study design or analytical purpose and appears descriptive rather than methodologically justified. Please revise.

Thank you, the irrelevant data has been removed and referenced in the introduction where appropriate to outline the rationale of the study.

16. The setting description is incomplete in methodological terms. It does not specify catchment population, inclusion pathways (e.g. community vs facility-based recruitment), or whether the setting is intended to be representative or convenience-based. Please revise.

The manuscript has been revised to clarify the recruitment approach as convenience-based. The recruitment strategy has been expanded to describe inclusion pathways, including community-based and facility-based recruitment. Additional detail has also been included to better characterise the study setting (lines 136-139).

17. The rationale for selecting the study setting is not explicitly integrated into the design justification and remains epidemiological narrative rather than methodological argumentation. Please revise.

The manuscript has been revised to clarify the rationale for the study setting and to strengthen its link to the study design, including provision of sufficient variability in immune status for diagnostic accuracy estimation and evaluation of test performance under real-world conditions. This has been addressed in the introduction rather than the trial design to avoid repetition (lines 102-118).

* Aims and objectives

18. The “Aims and objectives” section lacks clear alignment with the stated study design. The primary objective is appropriate for a diagnostic accuracy study; however, the secondary objectives are heterogeneous and extend beyond diagnostic accuracy (including acceptability, immunological correlation analyses, and multi-pathogen antibody profiling). These are not clearly justified within the described study design and appear methodologically disparate. Please revise.

    The objectives are not prioritised or structured according to primary and secondary endpoints, and there is no indication of how each objective will be operationalised within the study design framework. Please revise.

    Overall, the section requires restructuring to separate study design, setting justification, and objectives, and to ensure alignment between stated design, data collection framework, and planned analyses.

Thank you, the “Aims and objectives” section has been revised to improve structure and alignment with the study design. The primary objective has been refined to focus on diagnostic accuracy. Secondary objectives have been clarified and justified in the study rationale and streamlined to reflect exploratory analyses of acceptability, immunological relationships, and broader test performance, and are now more clearly aligned with the study’s design (lines 140-153).

* Study interventions

19. The heading “Study interventions” is misleading, as the study does not involve an intervention in the clinical trial sense. The content instead describes consent procedures, baseline data collection, and sample handling, which should be relocated to a “Study procedures” or “Data collection” section.

This section has been renamed to “Study procedures” (line 183).

20. "using the latest RNEC-approved" - define RNEC

Thank you for pointing out this omission, RNEC has been defined (line 185).

21. The statement that “a participant is enrolled at the point of sample provision” is imprecise and does not define eligibility confirmation, consent completion, or inclusion criteria in operational terms.

The manuscript has been revised to clarify the enrolment process, explicitly defining that participants are enrolled following completion of informed consent procedures and confirmation of eligibility according to predefined inclusion and exclusion criteria, prior to sample collection (lines 193-194).

* Saliva collection, processing and reporting of index test results

22. The description of the index test procedure is overly narrative and insufficiently standardised. Key operational details are missing, including swab type specification, storage conditions if needed, and time limits between collection, processing, and test initiation.

The description of the index test procedure has been revised to improve clarity and standardisation. A stepwise figure has been included to reduce narrative description. Additional operational details have been incorporated, including specification of the swab type (JSHXRT swab, Jiangsu HXRT MD Co., Ltd., China) (line 203), relevant storage conditions (line 209), and defined timing between sample collection and test initiation (line 220).

23. The pre-analytical instructions (“no eating or drinking except water for 30 minutes”) are stated without justification or reference to standardisation criteria, and it is unclear whether adherence will be monitored.

The manuscript has been revised to justify the pre-analytical instruction to avoid eating or drinking (except water) for 30 minutes prior to sampling, in order to minimise variability in saliva composition and reduce potential contamination that may affect assay performance (lines 210-213). The protocol now also specifies that adherence will be confirmed by participant self-report, and that sampling will be delayed if this criterion is not met (lines 210-211).

24. The saliva collection procedure is not fully specified in terms of training requirements for staff versus self-collection, nor is there clarification on whether both methods will be used interchangeably or under defined conditions.

The manuscript has been revised to specify training requirements for study staff and to clarify the conditions under which saliva samples will be self-collected or staff-collected (participant choice) (lines 213-214). It is now stated that both approaches will be conducted under supervision using a standardised procedure to ensure consistency across collection methods (Figure 1).

25. The interpretation process lacks adequate blinding procedures. Although dual scoring is described, there is no explicit statement regarding blinding of scorers to clinical data or reference standard results, nor is inter-rater reliability assessment planned or described.
    The statement that participants will be blinded to LFT results is methodologically unclear, as it is not explained how blinding will be maintained in practice, particularly given immediate interpretation at point of care.

    The blinding statement is insufficiently detailed, as it does not clarify whether laboratory personnel are also blinded to clinical data and index test results beyond the stated assessor role.

The manuscript has been revised to strengthen the description of the blinding process (lines 239-244). Assessment of inter-rater agreement has been incorporated using appropriate statistical measures.

26. The use of a semi-quantitative 0–10 intensity scale is introduced without validation, calibration procedures, or explanation of how this variable will be used in analysis, raising concerns about measurement validity and reproducibility.

Semi-quantitative scoring will be performed using a standardised reference card. Staff will be trained on use of the card. An outline of the training and an example of the reference card have now been included in the manuscript (Figure 1). The secondary objectives (lines 148-149) and statistical analysis plan (lines 321-324) have been revised to clarify the role of the semi-quantitative intensity scale. This measure is used for exploratory purposes only and is not intended as a validated quantitative outcome.

* Laboratory analysis plan

The laboratory analysis plan lacks sufficient methodological detail to ensure

reproducibility and clarity in a diagnostic accuracy protocol.

27. The designation of "multiplex bead-based assay and ELISA" as reference standards is not adequately justified or operationalised. It is unclear whether both assays will be used independently, in parallel, or hierarchically, and no algorithm is provided for resolving discordant results. The reference standard definition therefore remains ambiguous.

Thank you for this feedback. In the application for ethical approval, we planned for tetanus IgG concentration to be measured by either Luminex or ELISA. Given the wider access to the ELISA, which is a commercial test by the Binding Site, it has now been decided that this would be the most appropriate reference test. The Luminex assay is an in-house assay(1) that requires specific Luminex analysers and is not widely accessible. This has now been clearly defined in the manuscript, with reference to the Luminex assay removed (line 234).

28. The description of serum processing (clotting, extraction, heat-inactivation) is incomplete in key procedural aspects, including timing, centrifugation parameters, storage temperatures, and chain-of-custody procedures prior to shipment.

These procedural aspects have been added to the serum processing section (lines 221-226).

29. The shipping process to the University of Birmingham is stated without specifying transport conditions (e.g. temperature control, duration limits, quality assurance procedures), which is critical for antibody stability.

Serum samples will be shipped to the University of Birmingham Clinical Immunology Services on dry ice (UN1845) using an IATA-compliant triple-packaging system. Shipments will be maintained at ≤−20 °C throughout transit, with sufficient dry ice to ensure temperature stability for the full transport duration plus a 24-hour contingency. All packages will be labelled according to IATA PI650/PI954 requirements and transported via a certified cold-chain courier operating validated quality-assurance procedures (lines 228-232).

30. The inclusion of additional antibody testing for other vaccine antigens is described without clear linkage to predefined objectives or statistical analysis plans, raising concerns about exploratory data collection without analytical framework.

The manuscript has been revised to clarify the purpose of additional antibody testing against other vaccine antigens (lines 73-79). This component is now explicitly linked to a predefined secondary objective (lines 152-153) and a corresponding exploratory analysis plan (lines 332-336), including the use of positive and negative percent agreement. These analyses are clearly identified and not powered for definitive statistical conclusions.

* Sample storage

31. The term “pseudo-anonymisation” is used inconsistently with standard research governance terminology, and no clear description of coding, re-identification safeguards, or data linkage procedures is provided.

The correct terminology has now been used. The data management section has been expanded to provide a detailed description of pseudonymisation procedures, including coding, secure storage and access to linkage keys, and safeguards governing re-identification (lines 347-363).

32. The duration of sample storage (5 years) is stated without justification or reference to regulatory or ethical requirements.

The manuscript has been revised to clarify the justification for the sample storage duration. The 5-year retention period is now explicitly described as proportionate to the study aims and consistent with standard research governance and ethical requirements (lines 245-247).

33. Consent options for secondary use of samples, including commercial use, are described without clarification of governance framework, oversight, or separation between research and commercial pathways.

The manuscript has been revised to clarify the governance framework for secondary use of samples, including commercial use. Separate consent options are now explicitly described. Additional detail has been included on oversight mechanisms, controlled access to samples, and the clear separation between academic research and commercial pathways, ensuring all use is consistent with the original consent provided (lines 186-190).

* Acceptability and feasibility assessment

34. This is insufficiently defined methodologically. The sample size (10–20 participants and 10–20 healthcare professionals) is arbitrary and not justified. Recruitment strategy, sampling method, and saturation criteria are not specified.

    Focus group structure lacks clarity regarding stratification, group composition, and whether participant and healthcare professional groups are analysed separately.

    The description of qualitative methods is limited and does not specify interview guide development, data coding framework, or analytical approach (e.g. thematic analysis framework).

    The role of the facilitator is described in general terms without specifying training standards, reflexivity procedures, or potential influence on data collection.

Audio recording and transcription are stated without mention of translation validation, data anonymisation procedures during transcription, or quality control of transcripts.

Thank you for this feedback. The qualitative methods section has been substantially revised to address these points. Additional detail has been included on the sampling strategy, recruitment approach (lines 136-139), and justification of the sample size (lines 250-254). The structure and composition of focus groups are now clearly described, including separate analysis of participant and healthcare professional data (lines 257-263).

The methodological description has been expanded to specify the theoretical framework of Interpretivism, inclusion of the interview guide, and analytical approach (lines 262-263, 313-317). Details on facilitator training and reflexivity procedures have also been incorporated (lines 258-261).

Finally, the processes for audio recording, transcription, translation, and quality assurance have been clarified, including validation procedures, anonymisation, and independent review of transcripts and translations (lines 263-268).

* Sample size

35. The derivation of the primary sample size is presented in a fragmented manner, with unclear separation between assumptions for sensitivity and specificity. The calculation logic is difficult to follow, and the link between statistical inputs (specificity, confidence limits, power) and the final sample size is not transparently justified.

The sample size section has been revised to improve clarity and transparency of the calculation. The derivation is now structured to explicitly show that the number of participants below the protective threshold (driven by specificity assumptions) determines the overall sample size through the assumed prevalence (Table 2). The distinction between assumptions for specificity (primary driver) and sensitivity (secondary, conditional on prevalence) has been clarified.

36. The use of an assumed 90% protection rate is not supported with justification specific to the study population, and its applicability across heterogeneous subgroups is not addressed.

    The estimation of 35 unprotected participants as the basis for specificity is insufficiently justified for robust precision requirements, and the rationale for selecting a lower confidence limit of 65% is not explained.

The manuscript has been revised to provide references for the assumptions underlying the sample size calculation (Table 2). There are limited data available to guide these estimates, which has been outlined in the limitations of the protocol.

The requirement for 35 participants below the protective threshold is based on achieving acceptable precision around the estimate of specificity using the exact binomial method (lines 72-276).

The minimum acceptable lower confidence limit of 65% was selected to reflect a pragmatic balance between feasibility and precision in this setting, recognising the limited availability of seronegative individuals. This assumption has now been clarified and its limitations acknowledged (lines 274-276).

37. The power calculation for sensitivity is described narratively rather than formally, and does not clearly specify whether it is powered independently or conditionally on the assumed prevalence distribution.

The manuscript has been revised to clarify the framework for sensitivity estimation. It is now explicitly stated that sensitivity is not powered independently but is conditional on the achieved prevalence of protective immunity determined by the sample size calculation for specificity (lines 285-291). The underlying assumptions and their relationship to precision of the sensitivity estimate have been clarified to improve transparency (Table 2).

38. The stratified recruitment plan across four groups is not integrated into the statistical justification. It is unclear how stratification contributes to achieving the required number of unprotected participants, and whether recruitment targets are fixed or adaptive.

The immunosuppressed group has been included due to higher levels of sero-unprotected participants (lines 282-283). Recruitment targets are fixed, this has now been outlined in the manuscript (lines 283-284).

39. The subgroup assumptions (e.g. ≥97% protection in children and young adults, 50–70% in immunosuppressed adults) are stated without supporting empirical justification or reference to data sources.

References for the subgroup seroprotection estimates have now been included (Table 2). These are based on data from East Africa with the exception of the immunosuppressed group. Data in this area are limited, which has now been included in the strengths and limitations of the study.

40. The statement that 42 participants will be below the protective threshold is inconsistently derived and does not reconcile clearly with earlier calculations based on 35 unprotected participants.

The target of 42 was calculated based on the margins of error in the estimated seroprotective proportions and a need for a minimum 35 sero-unprotected participants. This has been clarified in the manuscript (Table 2, lines 277, 282).

41. The total sample size of 390, including a 10% attrition allowance, is presented without specifying whether attrition is expected to affect both index and reference standard completeness equally, or how missing data will be handled analytically.

The manuscript has been revised to clarify that the attrition allowance accounts for missing or invalid data affecting completeness of both index and reference standard measurements, without assuming equal impact on each (lines 288-291). Management of missing data is now outlined in the statistical analysis plan (lines 343-345).

Overall, the sample size section lacks transparency, reproducibility, and clear linkage between prevalence assumptions, subgroup structure, and diagnostic accuracy precision requirements.

* Statistical analysis

42. The distinction between primary and exploratory analyses is not clearly operationalised, and no formal statistical framework is provided to define how exploratory subgroup analyses will be controlled for multiplicity or interpreted in relation to the primary endpoint.

Thank you, the manuscript has been updated to separate primary and secondary (exploratory) analyses. Subgroup analyses will be exploratory in nature, and no formal adjustment for multiple comparisons will be performed. Findings will be interpreted cautiously.

43. The description of categorical variable analysis is generic and does not specify exact statistical methods, confidence interval approaches, or handling of clustering across predefined study groups. The primary diagnostic accuracy measures (sensitivity, specificity, likelihood ratios) are listed without specification of calculation methods, confidence intervals, or threshold definitions for classification of index and reference tests.

Description of the primary analysis has been expanded and references for calculations for sensitivity, specificity, likelihood ratios and confidence intervals have been included (line 301-309), as well as the threshold definitions for both tests (lines 217 and 235).

44. There is no description of how indeterminate or invalid index test results will be handled in the analysis, nor whether analyses will follow an intention-to-diagnose framework.

Analysis will be based on the population with both index test and reference standard results available. The handling of indeterminate or invalid test results has been included in the manuscript (lines 302-304).

45. Secondary accuracy analyses extend beyond the stated primary diagnostic objective and include multiple antigen targets (diphtheria, HIB) without justification of statistical power, multiplicity control, or pre-specification of hypotheses.

Secondary analyses have now been explicitly described as exploratory, and the objectives have been updated and expanded to justify the analysis (lines 145-153, 311-312).

46. The use of "positive and negative percent agreement" is not clearly justified in relation to established diagnostic accuracy metrics, and its interpretation relative to sensitivity/specificity is not clarified.

The manuscript has been revised to clarify the use of positive and negative percent agreement (PPA and NPA) in relation to standard diagnostic accuracy metrics. It is now explicitly stated that these analyses are conducted as agreement measures rather than diagnostic accuracy evaluations, as no true reference standard exists for these additional antigen targets. The use PPA/NPA rather than sensitivity/specificity has been clarified, and their interpretation as measures of agreement rather than accuracy is now explicitly described (line 323-326).

47. The proposed correlation analysis between test line intensity and antibody concentration is not methodologically specified, including the choice of correlation metric, treatment of non-linear relationships, or any planned transformation or standardisation of variables. In addition, its validity is questionable given that line intensity is derived from subjective visual assessment by operators rather than an objective, instrument-based measurement, introducing potential inter- and intra-observer variability that is not accounted for in the current plan.

The manuscript has been revised to provide a clearer methodological specification of this analysis. Use of a reference card to score line intensity to reduce subjectivity has been included (Figure 1). The relationship between LFT line intensity and serum anti-tetanus IgG concentration is now assessed using Spearman's rank correlation, with line intensity treated as an ordinal variable (lines 319-322).

This analysis is explicitly defined as exploratory. Inter-reader agreement in intensity scoring will be formally assessed using percentage agreement.

48. The comparison with vaccination history is included without addressing known limitations of vaccination record accuracy or misclassification bias.

The manuscript has been revised to acknowledge the limitations of vaccination history as a comparator (lines 325-327).

49. The data management plan is largely procedural and lacks detail on data validation, audit trails, handling of missing data, and data cleaning procedures prior to analysis.

Thank you for this feedback. The data management plan has been revised to include both automated data validation procedures within REDCap and manual data cleaning processes (lines 354-358). Additional detail has been provided on audit trails to ensure traceability of data entry and modification, as well as procedures for identifying and handling missing data prior to analysis (lines 344, 358).

50. The description of pseudo-anonymisation is inconsistent with standard terminology and does not clarify whether re-identification is possible or how linkage keys are managed.

The process of pseudonymisation including linkage has been better described in the data management plan (lines 347-352).

51. No statistical software, versioning, or reproducibility framework is specified for analysis.

Statistical software and versions have been included in the statistical analysis plan (297-299).

52. Overall, the section lacks a coherent, pre-specified analytical strategy linking objectives to statistical methods, and includes multiple secondary analyses without clear prioritisation, adjustment for multiplicity, or defined inferential framework.

We thank the reviewer for this helpful comment. The Statistical Analysis Plan has been revised to improve clarity and coherence. Analyses are now explicitly linked to the study objectives, with a clear distinction between confirmatory (primary) and exploratory (secondary) analyses. Secondary analyses have been structured and prioritised, and a statement has been added clarifying that these are hypothesis-generating, with no formal adjustment for multiplicity. We hope the revised section provides a clearer, pre-specified analytical framework and improved transparency.

The protocol has clear potential but requires substantial rethinking and restructuring, with improved clarity and methodological detail, to make it adequately informative and reproducible for application by other researchers.

References

1. Whitelegg AM, Birtwistle J, Richter A, Campbell JP, Turner JE, Ahmed TM, et al. Measurement of antibodies to pneumococcal, meningococcal and haemophilus polysaccharides, and tetanus and diphtheria toxoids using a 19-plexed assay. J Immunol Methods. 2012;377(1-2):37-46.
